# Parametric modeling of cellular state transitions as measured with flow cytometry

**DOI:** 10.1186/1471-2105-13-S5-S5

**Published:** 2012-04-12

**Authors:** Hsiu J Ho, Tsung I Lin, Hannah H Chang, Steven B Haase, Sui Huang, Saumyadipta Pyne

**Affiliations:** 1 Department of Applied Mathematics and Institute of Statistics, National Chung Hsing University, Taichung 402, Taiwan; 2 Department of Public Health, China Medical University, Taichung 404, Taiwan; 3 Department of Pathology and Surgery, Children's Hospital Boston, Harvard Medical School, Boston, MA 02115, USA; 4 Program in Biophysics, Harvard University, Cambridge, MA 02139, USA; 5 MD-PhD Program, Harvard Medical School, Boston, Massachusetts 02115, USA; 6 Department of Biology, Duke University, Durham, North Carolina, USA; 7 Institute for Biocomplexity and Informatics, University of Calgary, Calgary, Alberta T2N 1N4, Canada; 8 Broad Institute of MIT and Harvard University, Cambridge, MA 02142, USA; 9 Department of Medical Oncology, Dana-Farber Cancer Institute, Harvard Medical School, Boston, MA 02115, USA

## Abstract

**Background:**

Gradual or sudden transitions among different states as exhibited by cell populations in a biological sample under particular conditions or stimuli can be detected and profiled by flow cytometric time course data. Often such temporal profiles contain features due to transient states that present unique modeling challenges. These could range from asymmetric non-Gaussian distributions to outliers and tail subpopulations, which need to be modeled with precision and rigor.

**Results:**

To ensure precision and rigor, we propose a parametric modeling framework StateProfiler based on finite mixtures of skew *t*-Normal distributions that are robust against non-Gaussian features caused by asymmetry and outliers in data. Further, we present in StateProfiler a new greedy EM algorithm for fast and optimal model selection. The parsimonious approach of our greedy algorithm allows us to detect the genuine dynamic variation in the key features as and when they appear in time course data. We also present a procedure to construct a well-fitted profile by merging any redundant model components in a way that minimizes change in entropy of the resulting model. This allows precise profiling of unusually shaped distributions and less well-separated features that may appear due to cellular heterogeneity even within clonal populations.

**Conclusions:**

By modeling flow cytometric data measured over time course and marker space with StateProfiler, specific parametric characteristics of cellular states can be identified. The parameters are then tested statistically for learning global and local patterns of spatio-temporal change. We applied StateProfiler to identify the temporal features of yeast cell cycle progression based on knockout of S-phase triggering cyclins Clb5 and Clb6, and then compared the S-phase delay phenotypes due to differential regulation of the two cyclins. We also used StateProfiler to construct the temporal profile of clonal divergence underlying lineage selection in mammalian hematopoietic progenitor cells.

## Background

Flow Cytometry is among the most widely used platforms in biomedical research and clinical labs. It is used for investigation of a wide variety of biological problems at single cell level. Classical applications of flow cytometry include quantitative measurements of DNA content and cell cycle progression [1]. It is also one of the key platforms for studying dynamic cellular properties such as differentiation, proliferation and apoptosis, especially in the contexts of stem cells and cancer [2]. Such applications make flow cytometry the ideal platform for the purpose of identifying and monitoring the myriad states and functions in different specimens that vary over time under particular conditions and stimuli.

Typically, a flow sample is stained with fluorescent dyes, possibly attached to antibodies, and per cell events such as the expression of a cell-surface marker or the DNA content are measured in terms of fluorescence intensity. The distribution of these events are then plotted or modeled statistically for identification of important features in the sample. While developments in computational cytomics have produced many useful analytical methods (e.g. [3]), several important problems have not yet been addressed adequately. One such issue involves precise parametric modeling of dynamic features in temporal profiles such that the model parameters can characterize the transition of the populations in a sample through different cellular states. Often simple statistics such as population mean or size can be imprecise in the presence of unusually shaped distributions and outliers in temporal profiles. The modeling scenario could be complicated further by the adoption of different trajectories by different subpopulations. Indeed a rigorous algorithm for modeling cellular state transitions can not only automate the traditionally manual approach, which is subjective and labor-intensive, but also extend it to increasingly complex and high-throughput experiments.

Many major cytometric studies have highlighted the importance of characterizing temporal profiles at single cell resolution for a variety of purposes such as cell cycle expression kinetics (e.g. [4,5]), pharmacodynamics (e.g. [6,7]), signaling alterations in specific subpopulations (e.g. [8,9]), dynamics of differentiation into distinct lineages (e.g. [10,11]), and so on. Clearly, mathematical formulation of a cellular state-space, and the transitions therein, can help us model a given collection of temporal flow cytometric profiles with the required rigor. Thereupon we can study the changes in features (say, in comparison with those in control profiles) and monitor trends in parametric detail. Precise probabilistic modeling of sample distributions at each stage can automatically reveal such dynamic features as emergence of a tail subpopulation or change in the skewness of a cluster that are statistically well-defined as well as biologically insightful [3].

Temporal profiling of cellular state transitions in flow data can, however, present unique modeling challenges. Often the transient states produce non-Gaussian features such as asymmetric or trailing subpopulations owing to rush or delay in progression from one state to another [5]. Intermediate states might also produce outliers that cannot be clearly distinguished from the more distinctive states. Moreover certain metastable states may appear only inconsistently in a given time course [11]. Often the transient features appear and disappear at the tails of the more prominent distributions, and may be hard to model via automation. Thus a framework that uses robust probabilistic density functions to model time course data may be the best way to represent the underlying state-space, and reveal any sudden or gradual transition therein. In terms of the distribution of events in a flow sample, characteristics of different states may be determined by variation in size (say, percentage of cells in a peak or cluster), location (such as mean or mode) or significance (peak density) of the model components. While traditionally such changes were detected with manual or non-parametric techniques, several model-based frameworks have recently been applied with success, e.g. [3,12-15].

Here we present StateProfiler, a new framework based on finite mixture models of skew *t*-Normal distributions (STNMIX) for statistical characterization of flow cytometric time course data. In particular, we present in StateProfiler a new greedy Expectation-Maximization (EM) algorithm for fitting our STNMIX model. The greedy EM algorithm starts with a minimum number of distributions (or *components*) and sequentially inserts a new component to the mixture until model convergence is achieved. This parsimonious approach allows us to detect the dynamic appearance (and disappearance) of transient features that are characteristic of many state transitions. In addition, intermediate states are known to produce spatial features in the form of distributions with unusual shapes or low separation, which can lead to overlapping components, and hence to an overestimated number of model components. For optimal model selection, we therefore also provide in StateProfiler a new procedure for merging skew *t*-Normal components that are significantly overlapping in the mixture such that the change in entropy of the resulting model is minimal. Besides profiling of unusually shaped distributions and less well-separated features, this allows StateProfiler to tackle cellular heterogeneity that exists even within clonal populations.

We applied StateProfiler to learn the temporal features of cell cycle progression in two mutant strains of budding yeast *Saccharomyces cerevisiae*. Based on knockout of S-phase triggering cyclins Clb5 and Clb6, we compared the S-phase delay phenotypes resulting from the differential regulation of the two cyclins. Also we used StateProfiler to construct the overall temporal profile of clonal divergence underlying lineage selection in mammalian hematopoietic progenitor EML cells. By comparing the fitted models at each time point, we observed a slow and non-montonic convergence of clonal outlier subpopulations to a final median state.

## Results and discussion

Temporal profiling with StateProfiler has several distinct advantages. First, the skew *t*-Normal mixture fitted to the data is defined by a probability density function (pdf). This function is well-defined at any resolution and can be visualized as a smooth profile, which is, unlike kernel-based non-parametric representations, not dependent on bandwidth specification. Importantly, the pdf rigorously specifies the significance of every feature, which allows us to detect the significant ones in the profile, while ignoring the ones which are not. StateProfiler bases its optimal modeling on 3 strategies: (1) to begin with, asymmetric and heavy-tailed STNMIX components model the data precisely even in the presence of outliers or skewed populations, further (2) the parsimonious fitting of the model with greedy EM yields accurately estimated components, and finally, (3) any redundant components are merged into a well-fitted output profile. By design, our STNMIX model is computationally faster to fit than the skew *t *mixture (STMIX) model [3,12,16,17] without sacrificing precision or rigor. Ho *et al*. [13] summarized the differences between the STMIX and STNMIX models and showed the implementation of the STNMIX model is generally much simpler and faster than that of STMIX model.

For temporal profiling, certain parameters of STNMIX model such as shape are uniquely suited to detect lagging or hastening trends in subpopulations (such as delay phenotypes in gene knockout experiments) that directly correspond to interesting cellular states and functions. Clearly this is neither possible with non-parametric representations nor using traditional parametric models based on Gaussian, *t *or other symmetric components [5]. Moreover, such shape or size parameters could be used to test for separability among components - i.e. to identify tendencies of subpopulations to move towards or away from each other without actually changing their mean locations. Parametric "snapshots" of such back-and-forth trends can shed light on the the discrete (switch-like) or continuous (spectrum-like) nature of the state transitions, leading to statistical observation of systems exhibiting multistable dynamics [10].

To illustrate some applications of StateProfiler, we analyzed two previously generated datasets for studying (a) cell division cycle and (b) cell differentiation in different species.

### Cell cycle profiling

We applied StateProfiler to identify the temporal features of budding yeast cell cycle progression based on knockout of S-phase triggering cyclins Clb5 and Clb6. In late G1-phase, while both Clb5 and Clb6 activate Cdc28p to promote initiation of DNA synthesis, the exact mechanisms and extents of regulating this transition from G1 to S phase are distinct for the two cyclins [4]. In particular, Clb5 knockout causes a more prominent S phase defect during cell cycle progression in yeast cells than Clb6 knockout. Since DNA replication happens in S phase, we studied the dynamics of transition from the start and end states corresponding of one and two copies of the chromosomes (respectively, G1 and G2-M phases) while passing through intermediate states corresponding to S phase delay in the mutants. Interestingly, while genetic mutations are long known to produce delay phenotypes in cell cycle progression, few algorithms prior to StateProfiler could model the lag in the DNA distributions with precision.

**Figure 1:**
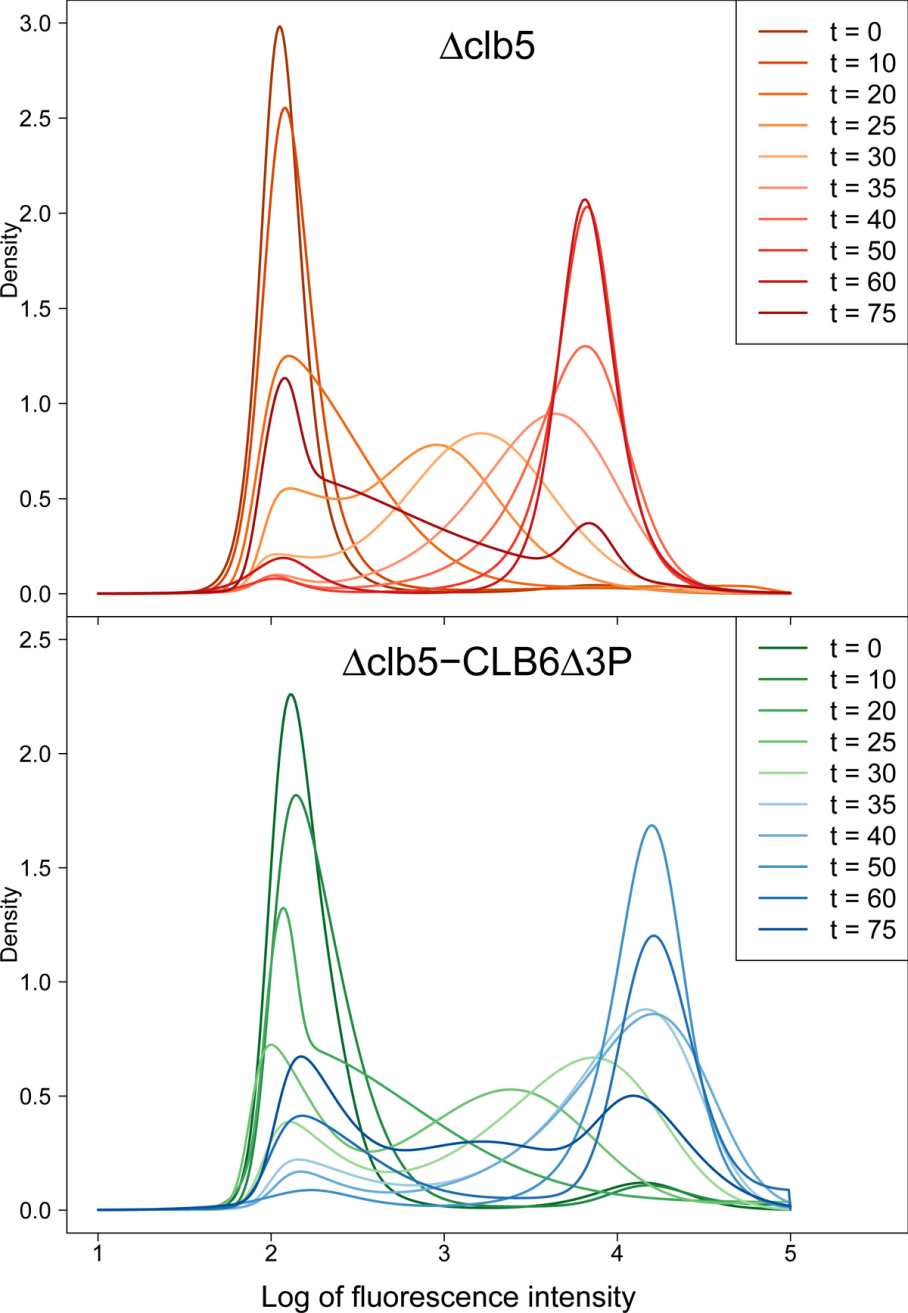
**Cell cycle time-course profiles**. Cell cycle time-course profiles. Overall spectrum of temporal profiles based on STNMIX modeling of flow cytometric DNA content data.

We fitted STNMIX models to flow samples from two cell cycle time courses with 10 time-points each in yeast cells with knockout of Clb5 (Clb5Δ) and Clb6 (Clb6Δ3P). The time courses spanned more than one cell cycle period with respect to wild-type yeast cells dividing under the same protocol. The fitted mixture models identified two or more components in every sample, which typically corresponded to the 1C and 2C peaks before and after DNA synthesis, along with subpopulations in the intermediate S-phase, thus characterizing an overall spectrum of profiles of different state transitions (Figure 1).

**Figure 2:**
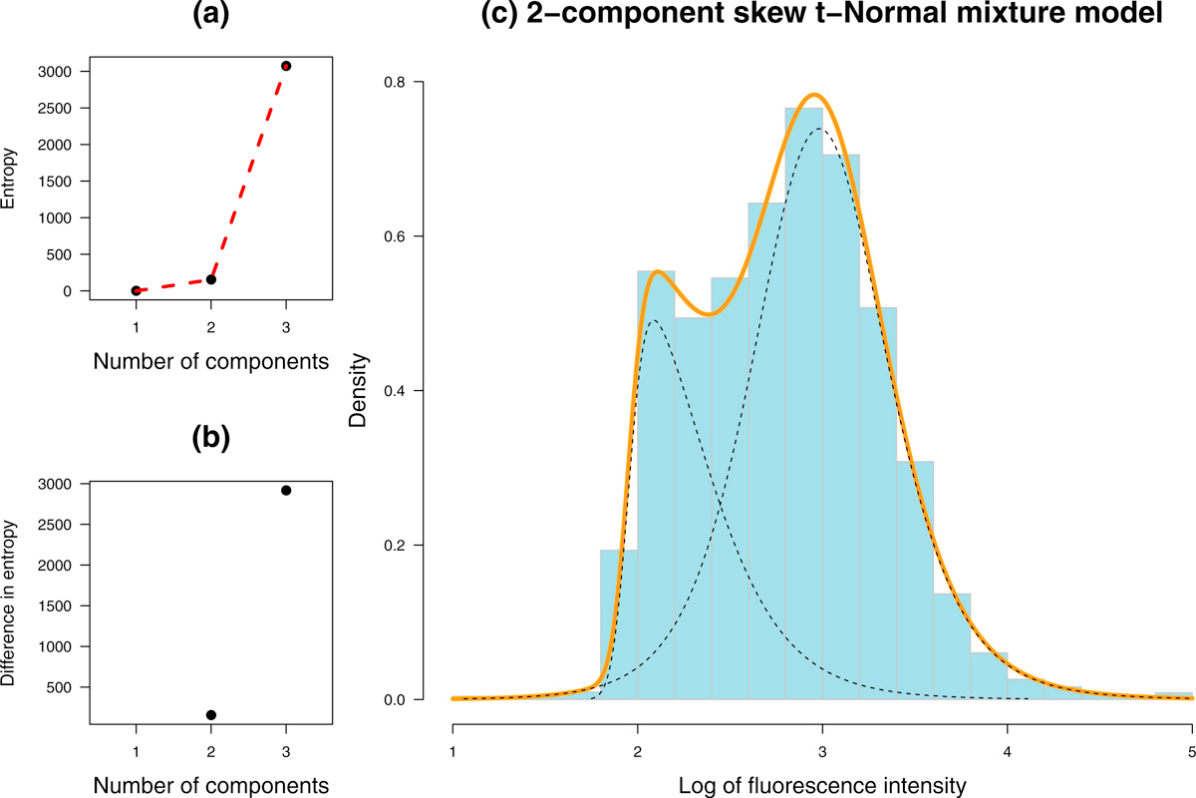
**Modeling a temporal flow cytometric profile**. Modeling a temporal flow cytometric profile. (a) Entropy values for a profile combining a given number of components (*g*) based on the results of the Greedy EM algorithm for Clb5Δ at *t *= 25 min. (b) Differences between successive entropy values as *g *increases. (c) DNA distribution for Clb5Δ at *t *= 25 min, as depicted by a histogram, is modeled with a 2-component skew *t*-Normal mixture. The orange curve shows the fitted profile while the underlying components are shown in dotted curves.

The smooth profiles of the noisy DNA histograms at every time-point are constructed with StateProfiler according to optimal change in the entropy values of the fitted model (Figure 2). For example, the entropy plot (Figure 2a, b) suggests a jump in entropy (or elbow) beyond *g *= 2 components for Clb5Δ data at *t *= 25 min (blue histogram in Figure 2c). The resulting 2-component profile is depicted by the orange curve in Figure 2c. The individual components involved in the model are identified and shown as black dotted curves. Their parameters could be used to detect features for purposes like sorting cells (FACS) or monitoring trends in specific subpopulations (e.g. note the lag in the left component in Figure 2).

**Figure 3:**
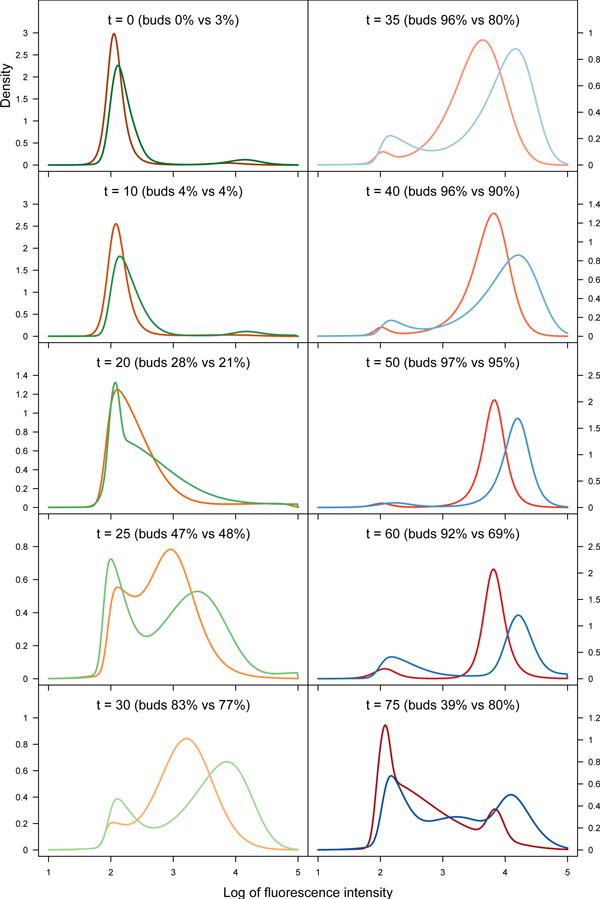
**Comparison of time-course profiles**. Comparison of time-course profiles constructed with StateProfiler. The orange-red and green-blue curves represent DNA distributions of Clb5Δ and Clb6Δ3P cells respectively. The time-points in minutes and budding information are indicated.

To determine the precision of STNMIX, we computed log-likelihood maxima ${\hat{\ell }}_{\text{max}}$, BIC values, and distances *D_n _*based on Kolmogorov-Smirnov (K-S) test statistic, and compared in Table 1 with the same for four competing 2-component mixture models (of normal, *t*, skew normal, and skew *t*) known from the literature [3,18]. According to BIC, the optimal selection of the STNMIX model with equal dfs is evident (e.g. the 2-component model at *t *= 25). As seen from *Dn*, we also conclude that STNMIX achieves the most precise modeling in terms of both the count and asymmetry of components in the given data. Further, we used the models for objective comparison of profiles both within and across time-courses. We computed the Gap statistic [19] as a measure of dispersion of cellular events between the two extreme states corresponding to the 1C and 2C peaks or clusters. Tested against a reference distribution of data with no clustering, the Gap statistics support the biological observation of Jackson *et al*. [4] that the Clb5 mutant shows more pronounced S-phase delay phenotypes than the Clb6 mutant and hence has less well-separated components in mid-cell cycle (e.g. *t *= 25). The contrast between the samples in terms of cells showing a slower state transition from 1C to 2C may be observed in Table 2 for different time-points. Finally, we observe the gradual variation in the key features at each successive time-point to gain insights into the differential regulation of the S-phase by the cyclins Clb5 and Clb6 (Figure 3).

**Table 1:** Details of competing models for Clb5 data

*t*	Criterion	NMIX	TMIX	SNMIX	STMIX	STNMIX
0	${\hat{\ell }}_{\text{max}}$	2539.78	2647.35	2682.44	2771.12	2759.40
	BIC	-5033.60	-5239.55	-5300.53	-5468.69*	-5445.25
	*D_n_*	0.0413	0.0262	0.0292	0.0164^†^	0.0185

10	${\hat{\ell }}_{\text{max}}$	1201.11	1224.87	1357.82	1405.31	1406.80
	BIC	-2356.27	-2394.60	-2651.32	-2737.09	-2740.08*
	*D_n_*	0.0424	0.0284	0.0312	0.0214	0.0190^†^

20	${\hat{\ell }}_{\text{max}}$	-5463.40	-5462.64	-4869.75	-4792.67	-4791.62
	BIC	10972.72	10980.37	9803.79	9658.80	9656.69*
	*D_n_*	0.0758	0.0715	0.0251^†^	0.0264	0.0266

25	${\hat{\ell }}_{\text{max}}$	-7040.90	-6981.07	-6992.27	-6918.17	-6916.52
	BIC	14127.73	14017.26	14048.84	13909.82	13906.53*
	*D_n_*	0.0147	0.0145	0.0155	0.0077	0.0075^†^

30	${\hat{\ell }}_{\text{max}}$	-7251.45	-7226.16	-7228.28	-7203.05	-7201.55
	BIC	14548.76	14507.34	14520.76	14479.46	14476.46*
	*D_n_*	0.0218	0.0175	0.0143	0.0129	0.0110^†^

35	${\hat{\ell }}_{\text{max}}$	-6413.58	-6412.58	-6374.92	-6320.38	-6334.20
	BIC	12872.96	12880.12	12813.96	12714.04*	12741.69
	*D_n_*	0.0196	0.0230	0.0136	0.0117^†^	0.0152

40	${\hat{\ell }}_{\text{max}}$	-4626.43	-4625.80	-4546.10	-4429.22	-4461.12
	BIC	9298.55	9306.44	9156.18	8931.56*	8995.37
	*D_n_*	0.0338	0.0306	0.0170	0.0123^†^	0.0184

50	${\hat{\ell }}_{\text{max}}$	-1286.86	-1121.26	-1286.53	-1093.80	-1086.34
	BIC	2619.40	2297.35	2637.03	2260.70	2245.79*
	*D_n_*	0.0222	0.0145	0.0218	0.0139	0.0132^†^

60	${\hat{\ell }}_{\text{max}}$	-2016.29	-1596.75	-1835.23	-1573.97	-1568.82
	BIC	4078.18	3248.21	3734.30	3220.89	3210.59*
	*D_n_*	0.0540	0.0203	0.0339	0.0172	0.0131^†^

75	$\hat{\ell_{\text{max}}}$	-8146.57	-7810.70	-7772.60	-7770.74	-7769.87
	BIC	16393.86	15731.29	15682.55*	15688.00	15686.25
	*D_n_*	0.0219	0.0119	0.0079^†^	0.0101	0.1104

Details of competing models for Clb5Δ data. Here, the notations stand for log-likelihood maxima ${\hat{\ell }}_{\text{max}}$max, BIC values, and distances *D_n _*based on Kolmogorov-Smirnov (K-S) test statistic. The abbreviation of models are the normal mixtures (NMIX), the *t *mixtures (TMIX), the skew-normal mixtures (SNMIX), the skew-*t *mixtures (STMIX) and the skew-*t*-normal mixtures (STNMIX), respectively. According to BIC and *D_n_*, the optimal selection of the STNMIX model with equal dfs is evident for most points. The smallest values of BIC and *D_n _*are indicated by * and ^†^, respectively.

**Table 2:** Measuring dispersion of events at each time point

Time	Gap1	Gap2	SE1	SE2
0	0.689	-0.170	0.016	0.016
10	0.436	-0.335	0.016	0.019
20	0.022	-1.245	0.012	0.016
25	0.203	-0.789	0.013	0.018
30	-0.338	-0.164	0.016	0.015
35	-0.439	-0.223	0.013	0.014
40	-0.371	-0.403	0.015	0.015
50	0.281	0.233	0.015	0.015
60	0.510	0.096	0.016	0.014
75	-1.550	0.100	0.013	0.014

Measuring dispersion of events at each time point. Gap statistics for Clb5Δ (Gap1) and Clb6Δ3P (Gap2) and associated standard errors.

### Cell differentiation profiling

Another key area in which flow data are extremely insightful about different state transitions is cell differentiation. In recent years, many important advances in biology have been made by studying the modes and mechanisms of differentiation especially in the context of stem cells and cancer. Stem cell differentiation has also been studied for their clinical applications such as in the field of regenerative medicine. An excellent review of the field is given in a recent text edited by Krishan *et al*. [2]. Over the course of differentiation, the profiles of expression of various markers - including those indicating stemness or commitment to a lineage - vary according to transitions of populations through unstable, metastable and eventually stable states. Often measurable phenotypic diversity appears due to cell-to-cell variability even within clonal populations, which are manifest and can be studied as outlier events or asymmetric or tail subpopulations. Sometimes these features are transient and peripheral, and could be hard to distinguish via automation. Accurate modeling of dynamic flow profiles is thus essential to identify or monitor transitional features as and when they appear (or disappear) for objective temporal characterization of the state-space components involved in differentiation.

In the present study, we analyzed clonal populations of EML cells, a multipotent mouse haematopoietic cell line that can differentiate into myeloid, erythroid, and other lineages. In a recent study, Chang *et al*. [11] measured the expression levels of the stem cell marker Sca-1 in different subpopulations of EML cells as time course data. They observed that cell-to-cell heterogeneity in this clonal progenitor population gave rise to Sca-1 outlier cells - cells that exhibit very high or low Sca-1 expression - and possessed distinct gene expression patterns. The heterogeneity could not be attributed to measurement noise or cell-cycle-dependent cell size variation. Eventually, however, each of these distinct Sca-1 subpopulations' profiles became similar to that of the median cells, thus revealing an attractor state. Yet it was noted [11] that the divergence lasted long enough to allow different propensities for either subpopulation, i.e. low and high Sca-1, to enter into a transient state that primes them for either the erythroid or the myeloid lineage, as captured by their differential expression of lineage-specific transcription factors.

For precise characterization of the dynamics by which population heterogeneity arose in this clonal population via outliers and subsided ultimately, cells with the lowest, middle and highest levels of Sca-1 expression were isolated by [11] using fluorescence-activated cell sorting (FACS). We call these subsets Sca-1^low^, Sca-1^mid^, and Sca-1^high^. Following FCAS, the sorted cells were immediately stripped of the staining antibody and cultured in standard growth medium. Subsequently, Sca-1 fluorescence intensity were measured individually for each of the 3 subpopulations as time course data. Similar measurements were made for an original clonal population of EML cells for comparison (we call it Sca-1^all^).

We applied the StateProfiler framework to model the flow profiles for 14-point time course data for each of the 4 populations. Often finite mixtures of Gaussians are used for modeling the theoretical subpopulation structure in such profiles [11,20]. However, using Gaussian components, precise modeling in the presence of outliers due to cell-to-cell heterogeneity is particularly difficult for clonal populations. This is because an optimal model must be able to accommodate such heterogeneity without requiring extra components, but Gaussian components with sharp tails are hardly robust against outliers. It leads to sub-optimal models with spurious subpopulations, which makes their biological interpretation difficult.

StateProfiler addressed the modeling problem in two ways. First, its skew *t*-Normal components are robust to outliers and asymmetry in the distributions. This helps in modeling transitional features even if they lead to unusually shaped or heavy tailed distributions. Second, even if redundant subpopulations were identified, the new merging procedure in StateProfiler can re-construct any significantly overlapping components in a statistically optimal fashion, i.e. to produce a combined profile by causing minimal change in entropy of the model pre- and post-reconstruction.

**Figure 4:**
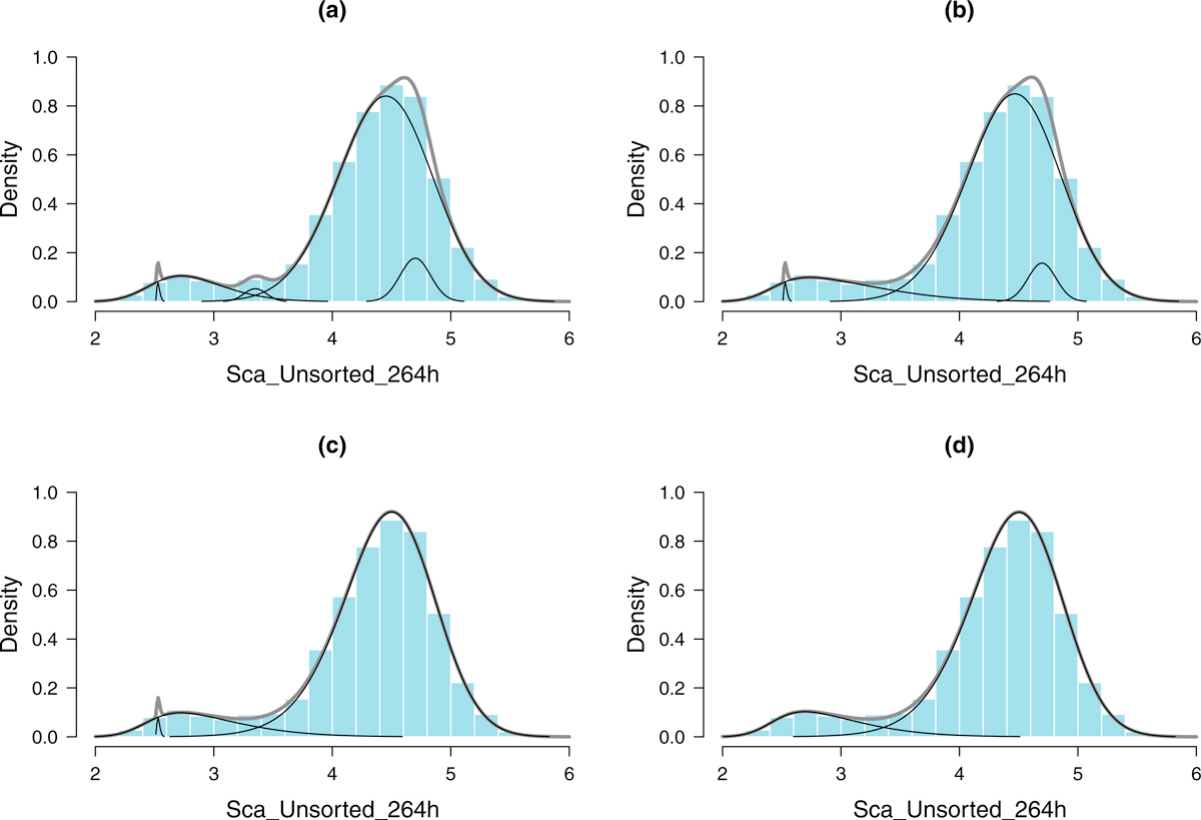
**An example of merging mixture components**. The Sca-1 expression data for the unsorted population of EML cells at 264 h is shown in the histogram. At each step of the merging algorithm, the fitted profile is shown as a thick grey curve, and the individual components in think black curves. (a) Initial profile computed by Greedy EM with *g *= 5, Entropy = 2351. (b) Merged profile with *g *= 4, Entropy = 573. Combining a group of components in the left significantly reduces entropy. (c) Merged profile with *g *= 3, Entropy = 297. (d) The final merged profile with *g *= 2 components and Entropy = 48.

**Figure 5:**
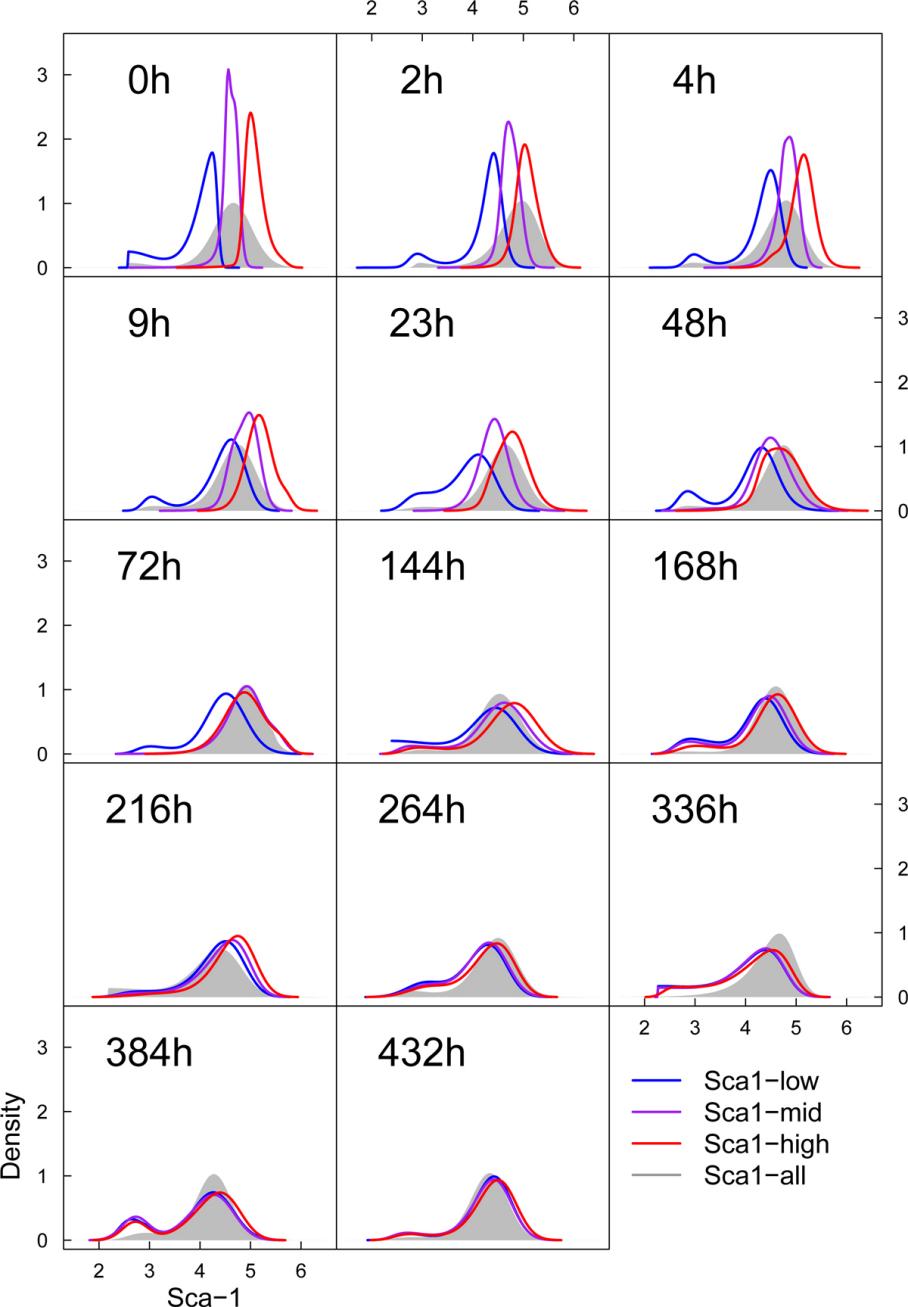
**Comparison of time-course profiles**. The temporal profiles of the 3 sorted subsets and the unsorted clonal population are constructed with StateProfiler, and plotted for visual comparison.

**Figure 6:**
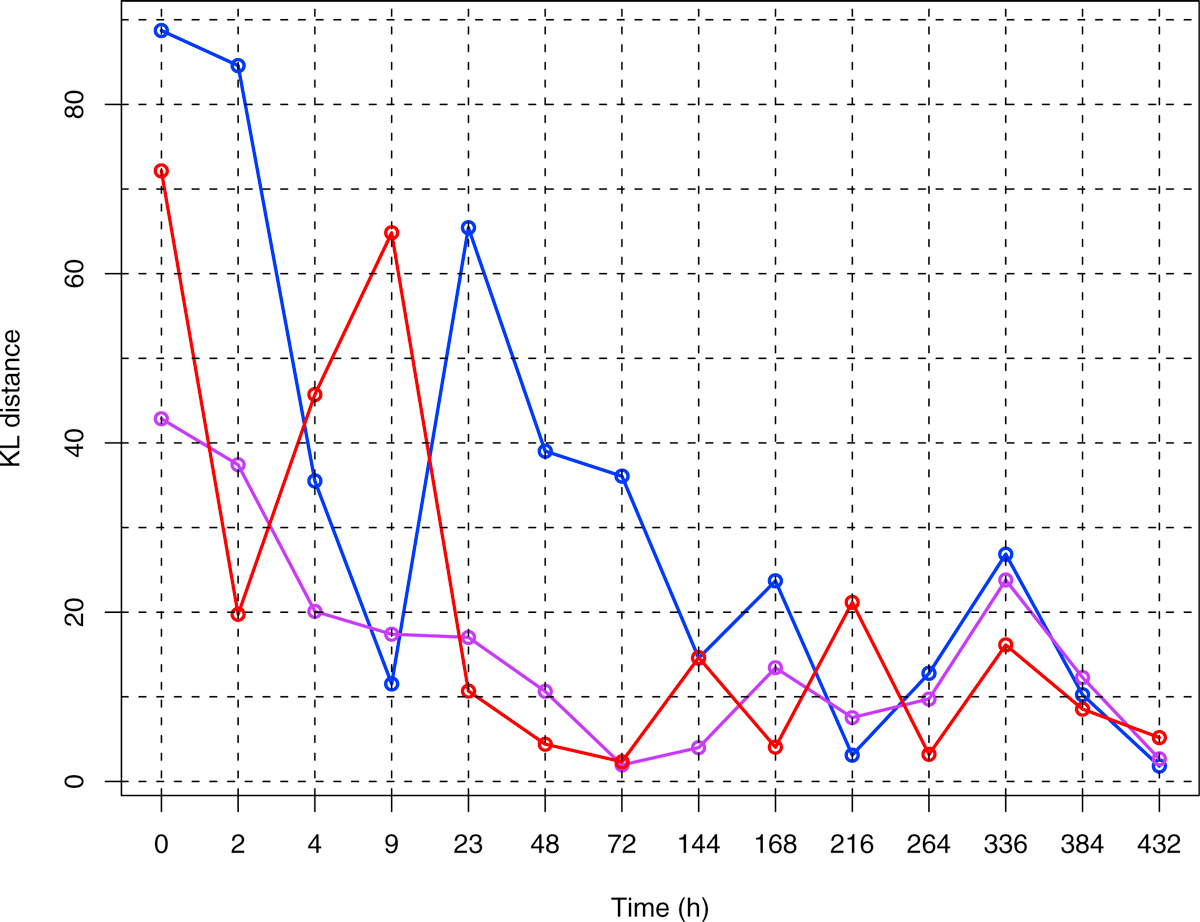
**The trend of convergence to the unsorted profile**. Kullback-Leibler (KL) distance of the profiles for each of the 3 sorted subpopulations from the unsorted profile at a given time-point. While the distances decrease with time, the trend is slow and does not appear to be monotonic.

The dual advantages of the StateProfiler modeling algorithm allowed us to compute highly accurate profiles of Sca-1 expression in the time course datasets for the three sorted and the unsorted EML cells. The steps of the merging procedure through which an optimal structure for the model is "stitched" together are illustrated with an example in Figure 4. Finally, we compared the divergence of the 3 sorted subsets from the corresponding unsorted population using Kullback-Leibler distances between the probability density functions specifying their profiles. A visual comparison of the profiles is shown in Figure 5. The trend of decreasing divergence, as the 3 sorted profiles become similar to the unsorted profile with progression of time, is shown in Figure 6.

StateProfiler's parametric characterization can reveal various features and trends of interest in terms of specific parameters. For instance, we observe that by 3 days, both Sca-1^mid ^and Sca-1^high ^have already started to resemble the unsorted population, and by 6 days, they actually have their own low Sca-1 tails. Another trend of possible interest is the slow but continuous fluctuation in the proportion of low Sca-1 outliers in the unsorted population. Finally, it appears that the eventual stable state when the 3 profiles finally coincide is reached at a point of time much later than 9 days, as suggested by [11], and takes probably double that time (432 h). In the mean time, as we see in Figure 6, the states might continue to drift closer and apart as in a dynamical system exhibiting multistable behaviour. If indeed the departure from the average state has biological functionality in the priming of cell fate commitment, then a non-monotonic, delayed restoration of the underlying molecular mechanisms may be justified by having more than a few cells with random fluctuation and call for further investigation.

## Conclusions

In this study, we described StateProfiler, a framework to construct temporal profiles with flow data, which can facilitate parametric modeling of cellular state transitions Towards this, we presented 3 key features of the framework. First, we described a finite mixture of skew *t*-Normal distributions. Second, we presented a new greedy EM algorithm for fast and optimal model selection. The parsimonious approach of our greedy algorithm allows us to detect the variation in the features as and when they appear and disappear at different points of time thereby offering a parametric characterization of the overall nature of state transition. Third, we designed a mixture merging procedure for ensuring robust estimation of the fitted profile. The code implementing the framework is available from the authors upon request. Indeed the proposed framework is effective, general and may be applied to other similar domains.

## Methods and materials

### Mixtures of skew Student-*t*-normal distributions

We describe the skew *t*-Normal mixture model (STNMIX) of StateProfiler. To simplify notation, we let *ϕ*(.) and Φ(.) denote the probability density function (pdf) and the cumulative distribution function (cdf) of the standard normal distribution, respectively. Let

$$t(x|\xi ,\sigma^{2},\nu )=\frac{\Gamma (\nu +1)/2)}{\Gamma (\nu /2)\sqrt{\pi \nu }\sigma }{\left(1+\frac{{\left(x-\xi \right)}^{2}}{\nu \sigma^{2}}\right)}^{-(\nu +1)/2}$$

denote the pdf of the *t *distribution with location *ξ*, scale *σ*^2 ^and degrees of freedom (df) *v*, and *t*(*x|v *) simply for the case when *ξ *= 0 and *σ *= 1; and let Γ(*α,β*) be the gamma distribution with density *g*(*x|α, β*) ∝ *x*^*α*-1^exp{-*βx*}. We start by defining the STN distribution and then note further properties.

As introduced by Gómez *et al*. [21], a random variable *Y *is said to follow the STN with location parameter *ξ *∈ ℝ, scale parameter *σ*^2 ^∈ (0, ∞), skewness parameter *λ *∈ ℝ and degrees of freedom *v *∈ (0,∞) it is has the density

(1) $$\psi \;\left(y\right)=2t\left(y|\xi ,\sigma^{2},v\right)\Phi \left(\lambda \frac{y-\xi }{\sigma }\right).$$

We shall write *Y~ STN*(*ξ*,*σ*^2^,*λ*,*v*) if *Y *has the density of (1).

Ho *et al*. [13] give following hierarchical representation of STN to establish an EM-type algorithm [22].

(2) $$\begin{array}{ccc} Y|\gamma ,\tau & \sim & N\left(\xi +\frac{\sigma \lambda }{\tau +\lambda^{2}}\gamma ,\frac{\sigma^{2}}{\tau +\lambda^{2}}\right),\\ \gamma |\tau & \sim & TN\left(0,\frac{\tau +\lambda^{2}}{\tau };\left(0,\infty \right)\right),\\ \tau & \sim & \Gamma \left(v/2,v/2\right), \end{array}$$

where *T N*(*µ, σ*^2^; (*a, b*)) represents the truncated normal distribution for *N*(*µ, σ*^2^) lying within the truncated interval (*a, b*).

Consider *n *independent random variables *Y*_1_*,..., Y_n_*, which are taken from a mixture of STN distributions. The pdf of a *g*-component STNMIX model is

(3) $$f\left(y_{j}|\Theta_{g}\right)=\overset{g}{\underset{i=1}{\sum }}w_{i}\psi \left(y_{j}|\theta_{i}\right),$$

Where *w_i_*'s are mixing proportions which are constrained to be positive and $\sum_{i=1}^{g}w_{i}=1,\psi \left(y_{j}|\theta_{i}\right)$is the STN density defined in (1) and Θ*_g_*= (*w*_1_,..., *w*_*g-*1_, *θ*_1_,..., *θ*_*g*_) represents all unknown parameters. Note that the component vector *θ*_*i *_consists of $\left(\xi_{i},\sigma_{i}^{2},\lambda_{i},v_{i}\right).$

Based on (2), a practical ECM/ECME algorithm [23,24] proceeds are described by Ho *et al*. [13] as follows:

**E-step: **Given $\Theta_{g}={\hat{\Theta }}_{g}^{\left(h\right)},$compute following $\hat{z_{ij}^{\left(h\right)}}$, ${\hat{\tau }}_{ij}^{\left(h\right)}$, $\hat{\kappa_{ij}^{\left(h\right)}}$ and ${\hat{\gamma_{1}}}_{\;\;ij}^{\left(h\right)}$ for *i *= 1,..., *g *and *j *= 1,..., *n*.

$$\begin{array}{c} {ẑ}_{ij}^{\left(h\right)}=\frac{{ŵ}_{i}^{\left(h\right)}\psi \left(y_{j}|{\hat{\theta }}_{i}^{\left(h\right)}\right)}{f\left(y_{j}|{\hat{\Theta }}^{\left(h\right)}\right)},\;{\hat{\tau }}_{ij}^{\left(h\right)}=\frac{{\hat{v}}_{i}^{\left(h\right)}+1}{{\hat{v}}_{i}^{\left(h\right)}+{\hat{u}}_{ij}^{2\left(h\right)}},\\ {\hat{\kappa }}_{ij}^{\left(h\right)}=\text{DG}\left(\frac{{{\hat{v}}_{i}}^{\left(h\right)}+1}{2}\right)-\text{log}\left(\frac{{\hat{v}}_{i}^{\left(h\right)}+{\hat{u}}_{ij}^{2\left(h\right)}}{2}\right),\\ {\hat{\gamma }}_{1ij}^{\left(h\right)}={\hat{\lambda }}_{i}^{\left(h\right)}{\hat{u}}_{ij}^{\left(h\right)}+\frac{\phi \left({\hat{\lambda }}_{i}^{\left(h\right)}{\hat{u}}_{ij}^{\left(h\right)}\right)}{\Phi \left({\hat{\lambda }}_{i}^{\left(h\right)}{\hat{u}}_{ij}^{\left(h\right)}\right)}, \end{array}$$

where ${\hat{u}}_{ij}^{\left(h\right)}=\left(y_{j}-{\hat{\xi }}_{i}^{\left(h\right)}\right)/{\hat{\sigma }}_{i}^{\left(h\right)}.$

**CM-step: **Update the estimation by

$$\begin{array}{c} {ŵ}_{i}^{\left(h+1\right)}={\hat{n}}_{i}^{\left(h\right)}/n,\\ {\hat{\xi }}_{i}^{\left(h+1\right)}=\frac{{\hat{b}}_{1i}^{\left(h\right)}+{\hat{\lambda }}_{i}^{2\left(h\right)}{\hat{b}}_{2i}^{\left(h\right)}-{\hat{\sigma }}_{i}^{\left(h\right)}{\hat{\lambda }}_{i}^{\left(h\right)}{\hat{b}}_{3i}^{\left(h\right)}}{\sum_{j=1}^{n}{ẑ}_{ij}^{\left(h\right)}{\hat{\tau }}_{ij}^{\left(h\right)}+{\hat{\lambda }}_{i}^{2\left(h\right)}{\hat{n}}_{i}^{\left(h\right)}},\\ {\hat{\sigma }}_{i}^{2\left(h+1\right)}\;=\;\frac{1}{{\hat{n}}_{i}^{\left(h\right)}}\overset{n}{\underset{j=1}{\sum }}{ẑ}_{ij}^{\left(h\right)}{\hat{\tau }}_{ij}^{\left(h\right)}{\left(y_{j}-{\hat{\xi }}_{i}^{\left(h+1\right)}\right)}^{2},\\ {\hat{\lambda }}_{i}^{\left(h+1\right)}=\frac{\sum_{j=1}^{n}{ẑ}_{ij}^{\left(h\right)}{\hat{\gamma }}_{1ij}^{\left(h\right)}{\hat{u}}_{ij}^{\left(h+1\right)}}{\sum_{j=1}^{n}{ẑ}_{ij}^{\left(h\right)}{\hat{u}}_{ij}^{2\left(h+1\right)}},\\ {\hat{v}}_{i}^{\left(h+1\right)}=\text{arg}\underset{v_{i}}{\text{max}}\left\{\frac{v_{i}}{2}\text{log}\left(\frac{v_{i}}{2}\right)-\text{log}\Gamma \left(\frac{v_{i}}{2}\right)+\left(\frac{v_{i}}{2}\right){\hat{b}}_{4i}^{\left(h\right)}\right\}, \end{array}$$

where ${\hat{n}}_{i}^{\left(h\right)}=\sum_{j=1}^{n}{ẑ}_{ij}^{\left(h\right)}$, ${\hat{b}}_{1i}^{\left(h\right)}=\sum_{j=1}^{n}{ẑ}_{ij}^{\left(h\right)}\;{\hat{\tau }}_{ij}^{\left(h\right)}y_{j}$, ${\hat{b}}_{2i}^{\left(h\right)}=\sum_{j=1}^{n}{ẑ}_{ij}^{\left(h\right)}y_{j}$, ${\hat{b}}_{3i}^{\left(h\right)}=\sum_{j=1}^{n}{ẑ}_{ij}^{\left(h\right)}\;{\hat{\gamma }}_{1ij}^{\left(h\right)}$, ${\hat{b}}_{4i}^{\left(h\right)}=\sum_{j=1}^{n}{ẑ}_{ij}^{\left(h\right)}\left({\hat{\kappa }}_{ij}^{\left(h\right)}-{\hat{\tau }}_{ij}^{\left(h\right)}\right)/{\hat{n}}_{i}^{\left(h\right)}$and ${\hat{u_{ij}}}^{\left(h+1\right)}=\left(y_{j}-\hat{\xi_{i}^{\left(h+1\right)}}\right)/\hat{\sigma_{i}^{\left(h+1\right)}}.$

If the dfs are assumed to be identical, say *v_1 _*= ⋯ = *v_g _*= *v*, we could update ${\hat{v}}^{\left(h\right)}$by

$$\begin{array}{l} {\hat{v}}^{\left(h+1\right)}\;=\;\arg\underset{\nu }{\max}\left\{\sum_{j=1}^{n}\log\{\sum_{i=1}^{g}{\hat{w}}_{i}^{\left(h+1\right)}\times \psi \;(y_{j}|{\hat{\xi }}_{i}^{\left(h+1\right)},{\hat{\sigma }}_{i}^{2(h+1)},{\hat{\lambda }}_{i}^{\left(h+1\right)},v)\}\right\}. \end{array}$$

The E-step and CM/CML-steps are alternately repeated until a suitable convergence rule is satisfied, e.g., the Aitken acceleration based stopping criterion $|\ell^{\left(h+1\right)}-\ell_{\infty }^{\left(h+1\right)}|<\varepsilon ,$where $\ell^{\left(h+1\right)}$is the observed log-likelihood evaluated at ${\hat{\Theta }}_{g}^{\left(h\right)},\ell_{\infty }^{\left(h+1\right)}$is the asymptotic estimate of the log-likelihood at iteration *h *+ 1 (see [18]; Chap. 4.9) and *ε *is the desired tolerance. For numerical analyses in this paper, a default value of *ε *= 10^-6 ^was used to terminate the iterations.

### Greedy learning for STN mixtures

In this section, we present a new greedy version of the EM algorithm to determine the optimum number of components in the fitting of STNMIX models. The greedy EM approach was first introduced by Vlassis and Likas [25]. The fundamental concept of the greedy EM algorithm is to start from a minimum number of components and sequentially insert a new component to the mixture until convergence is achieved. The stopping criterion can be a pre-specified maximum number of components or a pre-specified convergence tolerance.

Suppose a new component $\psi \left(y_{j}|\theta_{g+1}\right)$ is added to a *g*-component $f\left(y_{j}|\Theta_{g}\right)$. The resulting mixture takes the form of

$$f\left(y_{j}|\Theta_{g+1}\right)=\left(1-a\right)f\left(y_{j}|\Theta_{g}\right)+a\psi \left(y_{j}|\theta_{g+1}\right),$$

where 0 *< a <*1 and $\Theta_{g+1}=\left(\Theta_{g},\;a,\;\theta_{g+1}\right)$ with $\theta_{g+1}$being the added parameters $\left(\xi_{g+1},\;\sigma_{g+1}^{2},\;\lambda_{g+1},\;v_{g+1}\right).$ Given an old mixture $f\left(y_{j}|{\hat{\Theta }}_{g}\right)$, the weight *a *and $\theta_{g+1}$are optimally chosen to maximize the new log-likelihood

(4) $$\begin{array}{llll} L_{g+1} & =\overset{n}{\underset{j=1}{\sum }}\text{log}f\left(y_{j}|\Theta_{g+1}\right)\; & & \;\\ & =\sum_{j=1}^{n}\text{log}\left[\left(1-a\right)f\left(y_{j}|{\hat{\Theta }}_{g}\right)+a\psi \left(y_{j}|\theta_{g+1}\right)\right].\; & & \;\\ & \; & \end{array}$$

To find the optimal solution in (4), we start by performing a local search with for the newly inserted component. This gives rise to the following partial EM steps where $\tilde{\theta }$denotes and the partial ML estimates of *θ*. For notational simplicity, the subscript (*g *+ 1) is suppressed below in the Partial E-step.

**Partial E-step: **Calculating the conditional expectation of latent variables at the *k*th iteration, this yields

$$\begin{array}{llll} {\tilde{z}}_{j}^{\left(k\right)}\; & =\;\frac{{\tilde{a}}^{\left(k\right)}\psi \left(y_{j}|{\tilde{\theta }}^{\left(k\right)}\right)}{\left(1-{\tilde{a}}^{\left(k\right)}\right)f\left(y_{j}|\hat{\Theta_{g}^{\left(k\right)}}\right)+{\tilde{a}}^{\left(k\right)}\psi \left(y_{j}|{\tilde{\theta }}^{\left(k\right)}\right)},\; & & \;\\ \hat{\tau_{j}^{\left(k\right)}}\; & =\;\frac{{ṽ}^{\left(k\right)}+1}{{ṽ}^{\left(k\right)}+{ũ}_{j}^{2\left(k\right)}},\;{\tilde{\gamma }}_{1j}^{\left(k\right)}={\tilde{\lambda }}^{\left(k\right)}{ũ}_{j}^{\left(k\right)}+\frac{\phi \left({\tilde{\lambda }}^{\left(k\right)}{ũ}_{j}^{\left(k\right)}\right)}{\Phi \left({\tilde{\lambda }}^{\left(k\right)}{ũ}_{j}^{\left(k\right)}\right)},\; & & \;\\ {\tilde{\kappa }}_{j}^{\left(k\right)} & =\text{DG}\left(\frac{{ṽ}^{\left(k\right)}+1}{2}\right)-\text{log}\left(\frac{{ṽ}^{\left(k\right)}+{ũ}_{j}^{2\left(k\right)}}{2}\right),\; & & \;\\ & \; & \end{array}$$

Where ${ũ}_{j}^{\left(k\right)}=\left(y_{j}-{\tilde{\xi }}^{\left(k\right)}\right)/{\tilde{\sigma }}^{\left(k\right)}.$

**Partial M-step: **Updating the new parameters in (*a*, ***θ***_*g*+1_), we get

$$\begin{array}{c} {\tilde{a}}^{\left(k+1\right)}=\frac{\sum_{j=1}^{n}{\tilde{z}}_{j}^{\left(k\right)}}{n},\\ {\tilde{\xi }}_{g+1}^{\left(k+1\right)}=\frac{{\tilde{b}}_{1}^{\left(k\right)}+{\tilde{\lambda }}^{2\left(k\right)}{\tilde{b}}_{2}^{\left(k\right)}-{\tilde{\sigma }}^{\left(k\right)}{\tilde{\lambda }}^{\left(k\right)}{\tilde{b}}_{3}^{\left(k\right)}}{\sum_{j=1}^{n}{\tilde{z}}_{j}^{\left(k\right)}{\tilde{\tau }}_{j}^{\left(k\right)}+{\tilde{\lambda }}^{2\left(k\right)}\sum_{j=1}^{n}{\tilde{z}}_{j}^{\left(k\right)}},\\ {\tilde{\sigma }}_{g+1}^{2\left(k+1\right)}=\frac{\sum_{j=1}^{n}{\tilde{z}}_{j}^{\left(k\right)}{\tilde{\tau }}_{j}^{\left(k\right)}{\left(y_{j}-{\tilde{\xi }}^{\left(k+1\right)}\right)}^{2}}{\sum_{j=1}^{n}{\tilde{z}}_{j}^{\left(k\right)}},\\ {\tilde{\lambda }}_{g+1}^{\left(k+1\right)}=\frac{\sum_{j=1}^{n}{\tilde{z}}_{j}^{\left(k\right)}{\tilde{\gamma }}_{1j}^{\left(k\right)}{ũ}_{j}^{\left(k+1\right)}}{\sum_{j=1}^{n}{\tilde{z}}_{j}^{\left(k\right)}{ũ}_{j}^{2\left(k+1\right)}},\\ {\tilde{\nu }}_{g+1}^{\left(k+1\right)}=\underset{\nu }{\text{arg}\text{max}}\left\{\frac{\nu }{\text{2}}\text{log}\left(\frac{\nu }{\text{2}}\right)\right)\\ \left(\;\;\;-\text{log}\Gamma \left(\frac{\nu }{\text{2}}\right)+\frac{\nu }{2}{\tilde{b}}_{4}^{\left(k\right)}\right\}, \end{array}$$

Where ${ũ}_{j}^{\left(k+1\right)}=\left(y_{j}-{\tilde{\xi }}^{\left(k+1\right)}\right)/{\tilde{\sigma }}^{\left(k+1\right)}$, ${\tilde{b}}_{1}^{\left(k\right)}=\sum_{j=1}^{n}{\tilde{z}}_{j}^{\left(k\right)}{\tilde{\tau }}_{j}^{\left(k\right)}y_{j}$, ${\tilde{b}}_{2}^{\left(k\right)}=\sum_{j=1}^{n}{\tilde{z}}_{j}^{\left(k\right)}y_{j}$, ${\tilde{b}}_{3}^{\left(k\right)}=\sum_{j=1}^{n}{\tilde{z}}_{j}^{\left(k\right)}{\tilde{\gamma }}_{1j}^{\left(k\right)}$, and ${\tilde{b}}_{4}^{\left(k\right)}=\sum_{j=1}^{n}{\tilde{z}}_{j}^{\left(k\right)}\left({\tilde{\kappa }}_{j}^{\left(k\right)}-{\tilde{\tau }}_{j}^{\left(k\right)}\right)/\sum_{j=1}^{n}{\tilde{z}}_{j}^{\left(k\right)}$.

The above partial EM steps constitute a fast and simple procedure to locally seek for the maximum of $L_{g+1}$. To our experience, this local search scheme is very sensitive the initialization of *a *and *ξ*_*g*+1_. Similar to Vlassis and Likas [25], we provided a global search strategy for extracting proper parameter initialization for *a *and $\xi_{g+1}^{\left(0\right)}$. By a second-order Taylor expansion for $L_{g+1}$, we obtain the following approximation:

(5) $${\hat{ℒ}}_{g+1}={ℒ}_{g+1}(a_{0})-\frac{{\left[{\dot{ℒ}}_{g+1}(a_{0})\right]}^{2}}{2{\ddot{ℒ}}_{g+1}(a_{0})},$$

where ${\dot{ℒ}}_{g+1}(a_{0})$ and ${\ddot{ℒ}}_{g+1}(a_{0})$ are the first and second derivatives of $L_{g+1}$ evaluated at *a *= *a*_0_. It can be deduced from (5) that a local maximum of $L_{g+1}$ around *a*_0 _= 0.5 is given by

(6) $$\begin{array}{llll} {\hat{L}}_{g+1} & =\overset{n}{\underset{j=1}{\sum }}\text{log}\left(\frac{f\left(y_{j}|{\hat{\Theta }}_{g}\right)+\psi \left(y_{j}|\theta_{g+1}\right)}{2}\right)\; & & \;\\ & \;\;\;\;+\frac{{\left[\sum_{j=1}^{n}\delta_{j}\left(\theta_{g+1}\right)\right]}^{2}}{2\sum_{j=1}^{n}\delta_{j}^{2}\left(\theta_{g+1}\right)}\; & & \;\\ & \; & \end{array}$$

with

$$\delta_{j}\left(\theta_{g+1}\right)=\frac{f\left(y_{j}|{\hat{\Theta }}_{g}\right)-\psi \left(y_{j}|\theta_{g+1}\right)}{f\left(y_{j}|{\hat{\Theta }}_{g}\right)+\psi \left(y_{j}|\theta_{g+1}\right)}.$$

So the the optimal value of *a *can be calculated as

(7) $$\hat{a}=\frac{1}{2}\left(1-\frac{\sum_{j=1}^{n}\delta_{j}(\theta_{g+1})}{\sum_{j=1}^{n}\delta_{{}_{j}}^{2}(\theta_{g+1})}\right).$$

Following the suggestion of Li and Barron [26], one may set $\hat{a}=0.5$ for *g *= 1 and $\hat{a}=2/(g+1)$ for *g *≥ 2 as a default recommendation when the estimated value (7) fall outside the range of (0, 1).

In our global search, a convenience choice of ${\tilde{\sigma }}_{g+1}^{2\left(0\right)}$ is *n*^-1/5 ^times half of the sample variance $s_{y}^{2}$ whereas ${\tilde{\lambda }}_{g+1}^{2\left(0\right)}$ and ${\tilde{\nu_{g+1}}}^{\text{2}\left(\text{0}\right)}$ are always fixed at 0 and 10, respectively. For the initial choice of *ξ*_*g*+1_, we search over the 5th, 10th, 15th, ⋯ 95th quantiles of ***y ***and set ${\tilde{\xi }}_{g+1}^{\left(0\right)}$ to the one that maximizes (6).

The implementation of the greedy EM algorithm is summarized below.

1. Start with *g *= 1 and compute the ML estimates of the single-component STNMIX model via the ECME algorithm.

2. If *g >*1, estimate **Θ***_g _*via the EM-type algorithms.

3. Perform a global search to find a proper initialization of *a *and *ξ*_*g*+1_.

4. Apply the partial EM-steps until convergence. For instance, $|{\hat{L}}_{g+1}^{\left(k\right)}/{\hat{L}}_{g+1}^{\left(k-1\right)}-1|\;<10^{-6}$.

5. If ${\hat{L}}_{g+1}\leq {\hat{L}}_{g}+m$ then terminate, where *m >*0 is a penalty term. Otherwise allocate the new component to the model and go to 2. Set *g *= *g *+ 1.

Given *r *candidates (we have 19 quantiles of sample), the time complexity of our greedy EM algorithm is *O*(*ngr*). If overall sample was considered as candidates in the global search, then the running time is similar to Vlassis and Likas [25].

### Merging mixture algorithm

The greedy EM algorithm provides a convenient method for automatically selecting a number of components for a mixture model under reasonable assumptions (such as convexity of components). Yet if data have certain spatial features due to distributions with unusual shapes or low separation [8], it can lead to overlapping components, and hence to overestimation in the number of components in spite of the parsimonious approach. To augment our greedy algorithm for obtaining a robust estimate of the number of components, we extend the merging mixture approach of Baudry *et al*. [27] to skew *t*-Normal components. While merging techniques have been applied in the past to symmetric distributions [27,28], designing a procedure for asymmetric distributions obviates any need for spurious components that may be required for the sole purpose of modeling asymmetry, and thus avoids redundant merging.

The basic idea behind the procedure is to use the maximum merged entropy to iteratively combine two possibly overlapping clusters, until the result of combination belong a single cluster (see implementation in [28]). The steps of the merging algorithm in StateProfiler are described below.

1. Calculate the mean entropy of maximum estimation for *g *components as

$$\text{Ent}\left(g\right)=-\overset{n}{\underset{j=1}{\sum }}\overset{g}{\underset{i=1}{\sum }}{ẑ}_{ij}\text{log}{ẑ}_{ij}\geq 0,$$

where ${ẑ}_{ij}$ denotes the posterior probability given **Θ***_g _*fix at ${\hat{\Theta }}_{g}$.

2. Two clusters *l *and *l' *to be combined are those maximizing the criterion:

$$\begin{array}{c} \left(-\overset{n}{\underset{j=1}{\sum }}\left\{{ẑ}_{il}\text{log}{ẑ}_{il}+{ẑ}_{il^{\prime }}\text{log}{ẑ}_{il^{\prime }}\right\}\right)\\ \left(+\overset{n}{\underset{j=1}{\sum }}\left({ẑ}_{il}+{ẑ}_{il^{\prime }}\right)\text{log}\left({ẑ}_{il}+{ẑ}_{il^{\prime }}\right)\right) \end{array}$$

among all possible pairs of clusters (*l, l'*).

3. Obtain the merged entropy

$$\begin{array}{c} \text{Ent}\left(g-1\right)=-\overset{n}{\underset{j=1}{\sum }}\left\{\underset{i\neq l,l^{\prime }}{\sum }{ẑ}_{ij}\text{log}{ẑ}_{ij}\right)\\ \;\;\;\;\;\left(+{ẑ}_{i,l\cup l^{\prime }}\text{log}{ẑ}_{i,l\cup l^{\prime }}\right\}, \end{array}$$

where ${ẑ}_{i,l\cup l^{\prime }}={ẑ}_{il}+{ẑ}_{il^{\prime }}$ is the posterior probability of the new cluster *l *∪ *l'*.

4. Update $\hat{z_{j}}$ consists of the unmerged and merged posterior probabilities.

5. Set *g *= *g *- 1 and go to 2. Repeat until *g *= 1.

6. A solution of number of components can be identified (i) a sudden jump or "elbow" in a plot of the entropy of clustering versus the number of clusters, or (ii) peaks in a plot of the number of clusters versus the difference in entropy.

### Data and experiments

For details of the yeast cell cycle experiments and timecourse data analyzed by StateProfiler, see [4]. For details of EML cell differentiation data, see [11].

## Competing interests

The authors declare that they have no competing interests.

## Authors' contributions

HJH and TIL co-developed the statistical methods and performed data analysis. SP conceived the project, designed the approach, and analyzed the results. All authors contributed to the development of the methodology and to writing the manuscript. HJH and TIL contributed equally and are the first authors as well as listed in alphabetical order.
